# Cell-Specific Detection of miR-375 Downregulation for Predicting the Prognosis of Esophageal Squamous Cell Carcinoma by miRNA In Situ Hybridization

**DOI:** 10.1371/journal.pone.0053582

**Published:** 2013-01-03

**Authors:** Jiangchao Li, Xiaodong Li, Yan Li, Hong Yang, Lijing Wang, Yanru Qin, Haibo Liu, Li Fu, Xin-Yuan Guan

**Affiliations:** 1 State Key Laboratory of Oncology in Southern China, Sun Yat-Sen University Cancer Center, Guangzhou, China; 2 Vascular Biology Research Institute, Guangdong Pharmaceutical University, Guangzhou, China; 3 Department of Thoracic Surgery, Sun Yat-Sen University Cancer Center, Guangzhou, China; 4 Department of Oncology, The First Affiliated Hospital, Zhengzhou University, Zhengzhou, China; 5 Department of Clinical Oncology, The University of Hong Kong, Hong Kong, China; Vanderbilt University Medical Center, United States of America

## Abstract

MicroRNAs (miRNAs) play important roles in the regulation of genes associated with cancer development and progression. By the more deeply characterization of miRNAs’ effect in cancer development, it requires a useful tool to investigate expression and distribution of a miRNA in cancer cells and tissues. To fulfill this application demand, we developed a miRNA *in situ* hybridization (MISH) approach using the 2′-Fluoro modified miRNA probe in combination with enzyme-labeled fluorescence (ELF) signal amplification approach. MISH was used to study expression of miR-375 in esophageal squamous cell carcinoma (ESCC) cell lines and tissues using a tissue microarray (TMA) containing 300 cases. The results showed that our MISH approach is a practical way to detect expression and distribution of a tested miRNA in both cultured cells and archive tissue sections. MISH results also showed that miR-375 was frequently downregulated in ESCCs, which was significantly associated with advanced clinical stage (p = 0.003) tumor metastasis (p = 0.04) and poor outcome (p = 0.04) of ESCC. Moreover, the accuracy of MISH results could be confirmed by QRT-PCR. Our results demonstrated that MISH is a useful and reliable tool to study miRNA expression in solid tumors. Downregulation of miR-375 can be used as a biomarker to predict the outcome of ESCC.

## Introduction

MicroRNAs (miRNAs) are short (approximately 22 nucleotides) non-coding RNA molecules that regulate gene expression [1]. Recently, miRNAs have been reported to be involved in the development and progression of solid tumors including lung cancer and esophageal squamous cell carcinoma (ESCC) [2]–[5]. Studies have shown that miRNAs can be used as specific biomarkers for cancers [6], [7], for example, miR-375 and miR-146b-5p can be used to predict the prognosis of patients with ESCC and lung cancer, respectively [5], [8]. In our previous study, miR-375 has been found to be frequently downregulated in ESCC that is significantly associated with advanced clinical stage, tumor metastasis and poor prognosis [5].

Currently, quantitative real-time PCR (QRT-PCR), northern blot analysis and microarray are most frequently used assays to determine miRNA expression levels. One major limitation of these assays is that they cannot accurately detect miRNA expression in individual cell. Another application limitation is that they cannot investigate the distribution of miRNA in paraffin sections, which are necessary for studying the correlation between clinicopathological features and the miRNA of interest, as well as mechanisms of miRNA action in cancer cell proliferation or apoptosis [9]. Therefore, it is imperative to develop a useful and reliable tool to investigate expression and distribution of a miRNA in cancer cells and tissues. In the present study, we developed a miRNA *in situ* hybridization (MISH) approach using the 2′-Fluoro modified miRNA (2′-F RNA) probe combined with an enzyme-labeled fluorescence (ELF) signal amplification technology. 2′-F RNA has been reported to increase melting temperature (T_m_) and subsequently enhance the hybridization stability [10]. The MISH signal can be effectively amplified with ELF approach. Our data demonstrated that the MISH approach was a very reliable tool to detect expression level and cell distribution of a given miRNA in cancer cells and paraffin-embedded tissue sections.

## Materials and Methods

### Ethics Statement

ESCC tissue specimens used in this study were approved by the Committees for Ethical Review of Research involving Human Subjects at Zhengzhou University (Approval ID: UW 11-043). Written informed consents for the original human work that produced the tissue samples were obtained.

### Cell Lines and Clinical Samples

Non-small cell lung cancer cell lines ACC212102 and SCC211441 were established by our lab [11]. ESCC cell lines KYSE140, KYSE180 and KYSE510, were obtained from DSMZ (Braunschweig, Germany), the German Resource Centre for Biological Material. ESCC cell line HKESC1 was kindly provided by Professor Srivastava (Department of Pathology, The University of Hong Kong, Hong Kong) [12]. The cells were cultured in DMEM with 10% FBS on glass cover slides at 37°C with 5% CO_2_. When the cell confluence reached 70%–80%, they were immediately fixed in 10% formalin for 2 hr, and subsequent ISH was performed on the glass cover slides.

The primary ESCC tumor tissues and paired nontumorous tissues were obtained from Linzhou Cancer Hospital (Henan, China). No patients recruited in this study have received any preoperative treatment.

### Tissue Microarray (TMA)

A total of 300 formalin-fixed and paraffin-embedded ESCC specimens and the corresponding normal esophageal tissues were obtained from the archives of diagnosed ESCCs in Linzhou Cancer Hospital (Henan, China). The TMA blocks were constructed according to the method described previously [13]. All tissue samples used in this study were approved by the Committees for Ethical Review of Research Involving Human Subjects at Zhengzhou University.

### MISH Probes

Oligonucleotide probes complementary to hsa-miR-146b-5p and has-miR-375 were purchased from the Exonbio Lab (Guangzhou, China). The probe sequences were as follows: 5′-AGCCTATGGAATTCAGTTCTCA-3′ (miR-146b-5p) and 5′-TCACGCGAGCCGAACGAACAAA-3′ (miR-375). These oligonucleotides contain 2′-fluoro-modified RNA residues (2′-F RNA) at the 3, 6, 15 and 20 base, which can increase melting temperature (T_m_) and subsequently enhance the hybridization stability. Both 5′ and 3′ ends were labeled by digoxin (DIG). A scramble probe 5′-AGTCTATGGTATTCAGTACTCA-3′ was used as a control.

### *In Situ* Hybridization

Approximately 5–10 µm thick sections from tissues or tissue microarray blocks were deparaffinized, dehydrated and subsequently immersed in 0.2 N HCl for 20 min. Slides were then immersed in 0.5% Tween (PBS) solution, and the tissues were fixed in 10% neutral-buffered formalin. Proteinase K (working solution: 200 µg/ml in PBS) digestion was used to treat fixed tissues at 37°C for 5 min, but culture samples on glass cover slides were treated only with 0.1% Triton-100/PBS. After digestion, slides were immersed in RNase-free water for 3 min and air dried. The slides were then prehybridized in hybridization buffer (65% formamide, 5×SSC, 1% Tween-20, 100 µg/ml yeast RNA) at 37°C for 2 hr, followed by the hybridization with probe at 37°C for 24 hr. After hybridization, slides were washed in 2×SSC with 0.5% Tween-20 two stringent times for 5 min at room temperature.

### MISH Detection

Enzyme-labeled fluorescence (ELF) signal amplification kit (Invitrogen, San Diego, CA) was used to amplify and detect MISH signals according to the manufacturer’s protocol [14]. The kit uses substrate cleavage by a phosphatase to produce a green fluorescence at the site of enzymatic activity. The MISH signal is up to 40 times brighter compared to probes directly labeled with fluorophores. The slides were counterstained with Hoechst 33342 (1 µg/ml). Images of miRNA signals in cells were captured by an Olympus BX51 fluorescence microscope. To make the MISH signals comparable among images, the exposure time for all image captured in the present study was 0.1 seconds.

### Quantitative Real-Time PCR of miRNA

Total RNA was extracted from cultured cells using the TRIzol reagent (Invitrogen, Carlsbad, CA). miRNAs from paraffin-embedded ESCC specimens were extracted using the miRNeasy FFPE kit (Qiagen, Valencia, CA). Advantage RT-PCR Kit (Clontech Laboratories, Inc., Mountain View, CA) was used to synthesize the first strand complementary DNA. qPCR was performed with primers for *miR-375* and 18 s as described previously with SYBR in 7900 HT Real-Time PCR System (Applied Biosystems, Carlsbad, CA) [5]. Expression level was normalized against endogenous 18 s for *miR-375*. Triplicate independent experiments were performed.

### Statistical Analysis

Statistical analysis was performed using Statistical Package for Social Sciences (SPSS) 16.0 for Windows (SPSS Inc., Chicago, IL, USA). χ^2^-test or Fisher’s exact test was used to analyze the association of *miR-375* expression and clinicopathological parameters. The survival curves were plotted by using Kaplan-Meier analysis. Differences were considered significant when p value was less than 0.05.

## Results

### Detection of miRNA Expression in Cell Lines and Tissue Sections

**Figure 1:**
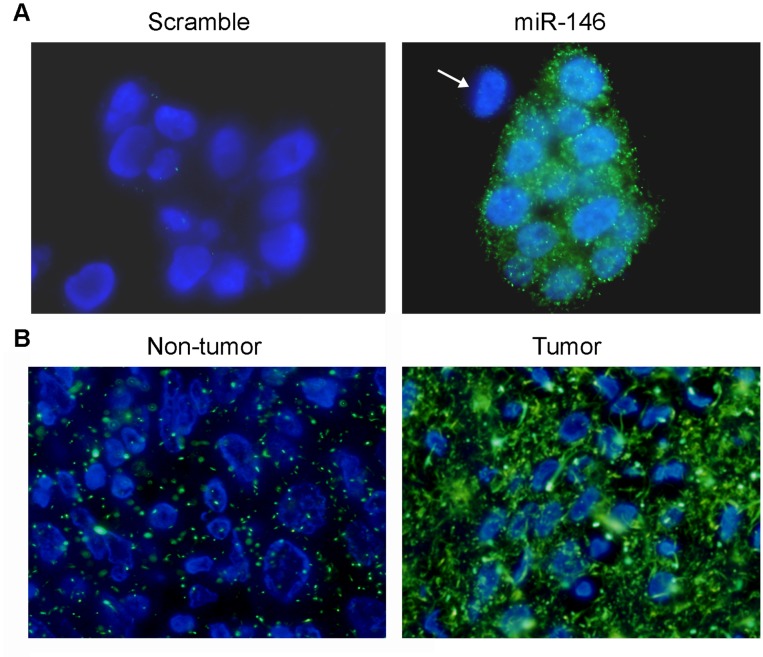
Detection of miR-146b-5p expression in lung cancer by MISH. (**A**) Representative images of miR-146b-5p expression in a lung cancer cell line SCC211441. Cytoplasmic expression of miR-146b-5p could be specifically detected by 2′-Fluoro modified miR-146b-5p probe but not by the scramble control probe (1,000× magnification). Notably, expression of miR-146b-5p was not observed in one tumor cell (right panel, indicated by an arrow). (**B**) MISH can be effectively used to detect miRNA expression in archive paraffin-embedded tissue sections. Overexpression of miR-146b-5p was detected in the cancer tissue by MISH compared to the corresponding non-tumor tissue (1,000× magnification).

**Figure 2:**
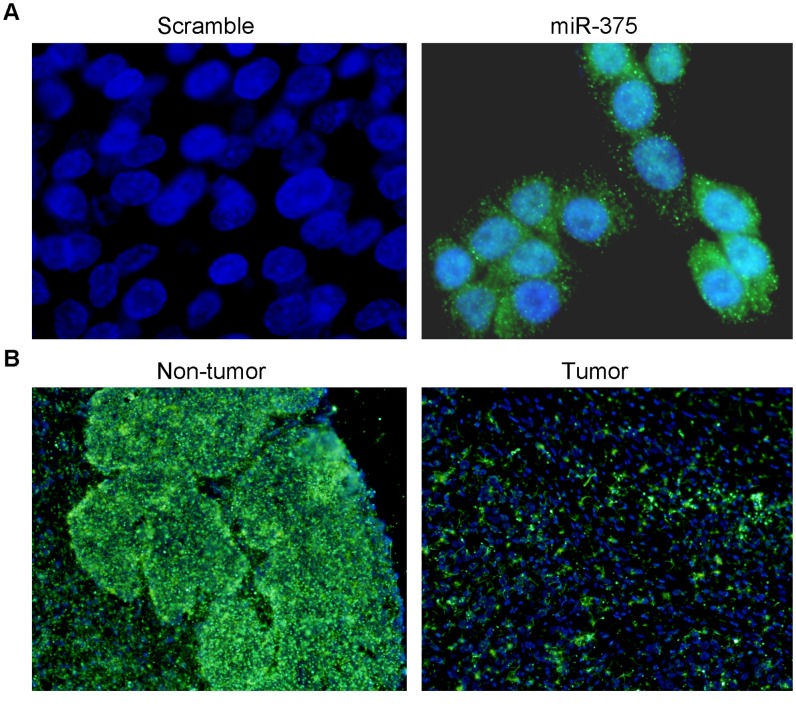
Detection of miR-375 expression in ESCC by MISH. (**A**) Expression of miR-375 in ESCC cell line KYSE-510 could be specifically detected by miR-375 probe but not by the scramble control probe (1,000× magnification). Notably, the expression level of miR-375 was various among tumor cells. (**B**) Representative images of miR-375 expression in a paired archive tissue sections (ESCC tumor and its corresponding non-tumor tissues) detected by MISH. The result showed that expression of miR-375 was downregulated in the cancer tissue compared to its corresponding non-tumor tissues (200× magnification).

To explore whether the MISH is a useful tool in miRNA detection, MISH was applied to study expression of miR-146b-5p in lung cancer cell lines and cancer tissues. In lung cancer cell line SCC211441, the fluorescence signals of miR-146b-5p probe (green color) were dispersed uniformly throughout the cytoplasm of tumor cells (Fig. 1A, right panel). Interestingly, no miR-146b-5p signals were observed in a few of the cells (Fig. 1A, indicated by an arrow). No specific signals could be detected in cells hybridized with scramble probe (Fig. 1A, left panel). In lung cancer tissue sections, overexpression of miR-146b-5p could be only detected in tumor tissue (Fig. 1B, right panel) but not in non-tumor tissue (Fig. 1B, left panel). Similar results could be observed in MISH with miR-375 probe in ESCC cell line and tissue section. In ESCC cell line KYSE510, expression of miR-375 could be detected by MISH with specific probe (Fig. 2A, right panel), but not by scramble probe (Fig. 2A, left panel). In ESCC tissue sections, strong expression of miR-375 could be detected in non-tumor tissue (Fig. 2B, left panel), but not in tumor tissue (Fig. 2B, right panel).

### Validation of MISH Results with QRT-PCR

**Figure 3:**
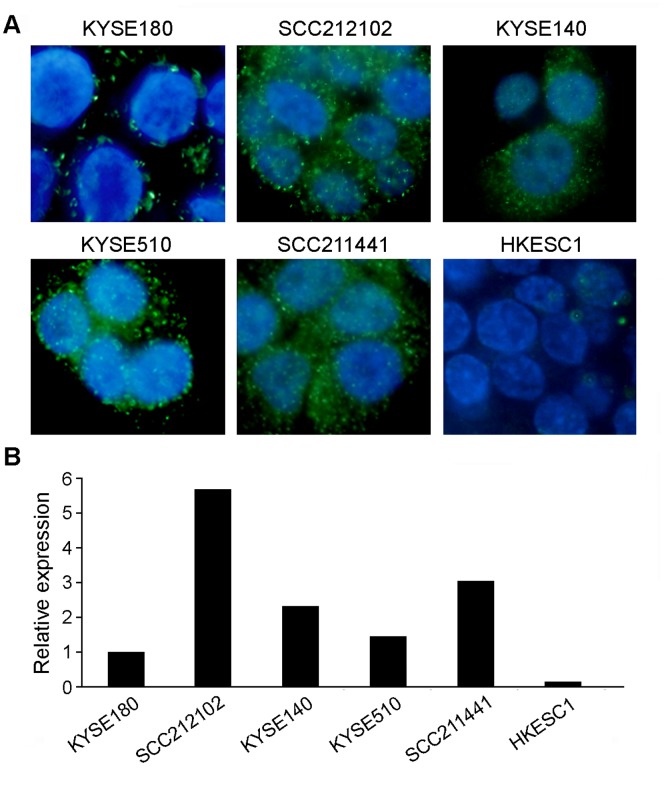
Validation of MISH results by RT-PCR. (**A**) Expression of miR375 was detected in a series of cell lines by MISH (1,000× magnification). The result showed that miR375 expression level was consistent with data derived from RT-PCR (**B**).

To validate the accuracy of the MISH, QRT-PCR was performed in multiple cell lines and the results were compared to the MISH results. The expression level of miR-375 was varied in different cell lines and high-level expression was detected in SCC212102, KYSE140, KYSE510 and SCC211441 (Fig. 3A). Consistent with MISH data, expression level of miR-375 was also higher in these cell lines (Fig. 3B), suggesting that MISH assay could accurately reflect the expression level of a tested miRNA.

### MiR-375 was Frequently Downregulated in ESCCs

**Figure 4:**
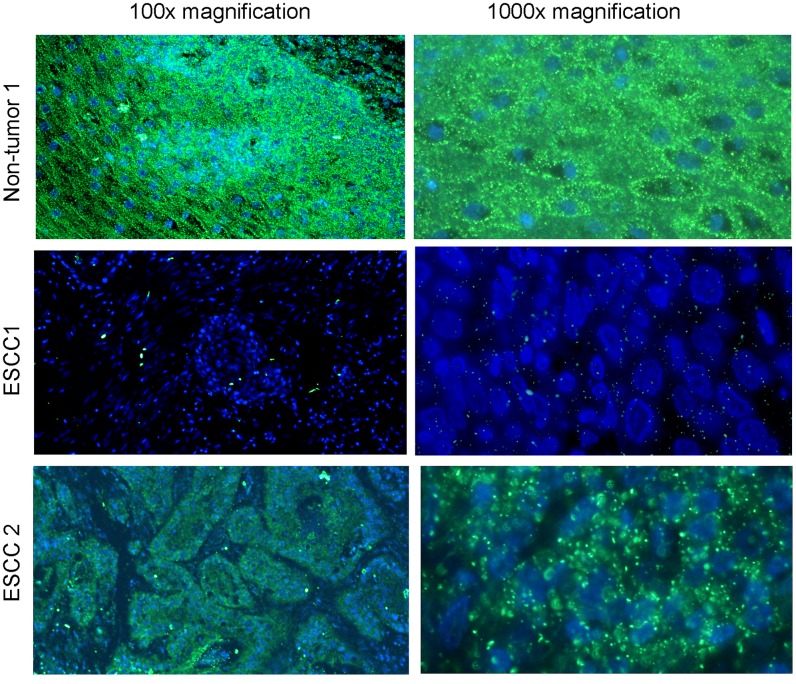
Detection of miR-375 expression in ESCCs by MISH using TMA. Representative images of MISH in one pair of ESCC (tumor vs non-tumor tissues) with miR-375 downregulation and one ESCC tumor specimen with expression of miR-375. Left: 100× magnification; Right: 1000× magnification.

In our previous study, we have demonstrated that miR-375 was frequently downregulated in ESCCs, which was significantly associated with advanced clinical stage, metastasis and poorer outcome of ESCC [5]. Here, we applied MISH to study the clinical significance of miR-375 downregulation in 300 pairs of primary ESCCs and corresponding non-tumor tissues using tissue microarray (TMA). Informative results were obtained from 249 pairs of ESCC cases. Non-informative samples included lost samples and samples with too few cells. MISH result showed that downregulation of miR-375 could be detected in 135/249 (54.2%) of informative ESCC tissues compared with their corresponding non-tumor tissues (Fig. 4). To validate our MISH results, qRT-PCR was used to compare expression levels of miR-375 between tumor and their corresponding non-tumor tissues in 10 randomly selected ESCCs (including 5 cases with downregulation of miR-375). The results showed that downregulation and normal expression of miR-375 was detected in 4/5 and 4/5 cases, respectively. This data suggests that MISH result is consistent with qRT-PCR result.

### Downregulation of miR-375 is Associated with Poor Outcome of ESCC

**Figure 5:**
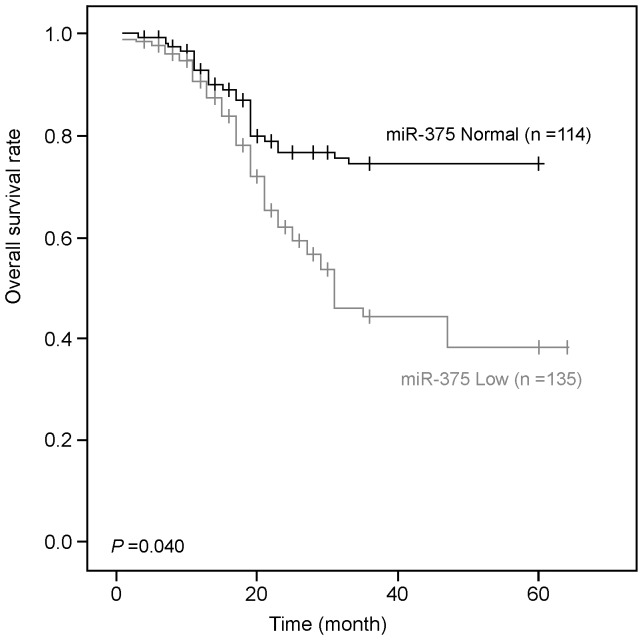
Kaplan-Meier analysis shows that downregulation of miR-375 expression was significantly associated with poor overall survival in the ESCC (p = 0.040, Log-rank test).

Clinical association study was applied to analyze the correlation of miR-375 downregulation with ESCC clinicopathological features using the SPSS 16.0 software. Downregulation of miR-375 was defined when its fluorescent signals in tumor tissue was obviously less and weaker than that in its corresponding non-tumor tissue. As shown in table 1, the low expression of miR-375 was significantly correlated with lymph node metastasis (p = 0.04) and advanced clinical stage (p = 0.003). Kaplan-Meier analysis was performed to further investigate the prognostic significance of miR-375 in ESCC. The result showed that downregulation of miR-375 was significantly associated with poorer overall survival rate in ESCC (Fig. 5).

**Table 1:** Clinical correlation of miR-375 expression in 249 of primary ESCCs.

Clinical Feature	Number	miR-375 expression		*P* value
		Low	Normal	
**Gender**				0.308
* Male*	136	78(57.4%)	58(42.6%)	
* Female*	113	57(50.4%)	56(49.6%)	
**Age**				0.609
* ≤60*	105	59(56.2%)	46(43.8%)	
* >60*	144	76(52.8%)	68(47.2%)	
**Tumor size (cm^3^)** a, b				0.295
* <10*	57	29 (50.9%)	28 (49.1%)	
* 10–20*	71	44 (62.0%)	27 (38.0%)	
* >20*	66	33 (50.0%)	33 (50.0%)	
**Tumor invasion** c				0.257
* T1*	19	7 (36.8%)	12 (63.2%)	
* T2*	73	39 (53.4%)	34 (46.6%)	
* T3*	157	89 (56.7%)	68 (43.3%)	
**Lymph node Metastasis**				**0.040**
* N0*	141	68 (48.2%)	73(51.8%)	
* N1*	108	67 (62.0%)	41 (38.0%)	
**TNM stage**				**0.003**
* I–II*	166	79 (47.6%)	87 (52.4%)	
* III–IV*	83	56 (67.5%)	27 (32.5%)	

a Tumor size was measured and obtained by 0.5×the length×(the wide)^2^.

b Partial data is not available, and the all statistic was based on the informative data.

c Invasion was defined by results on final pathological analysis.

## Discussion

Many miRNAs have been reported to serve as biomarkers to predict the overall survival of cancer patients, for example, miR-146b-5p has been applied to estimate the survival of lung cancer patients [8]. However, the methods of detecting miRNA, such as RT-PCR or northern blot assays, cannot accurately detect miRNA expression or localization in individual cell or in archival tissue specimens. This technical limitation has limited miRNA study. In studying correlation between a given miRNA and cancer microenvironment, for example, we need to distinguish miRNA expression levels in various cell types, such as fibroblasts, immunocytes or cancer cells. To fulfill this increasing demand, we developed a MISH approach to detect miRNA expression in both cell lines and cancer tissue sections. Previous study by Neely et al has found that fluorescent locked nucleic acid (LNA) probe, which increased the stability of binding to the target miRNA, could be used to detect expression level of miRNA [14]. Similarly, our study found that the 2-F modified probes could also increase the miRNA binding stability. Combining 2′-F RNA and ELF signal amplification technologies, we found MISH is able to provide strong and specific signals for evaluating the expression level and distribution of the tested miRNA in both cultured cells and archival tissue specimens.

Several approaches have been used to quantify miRNA expression. For example, fluorescent-labeled LNA probe has been applied to quantify the expression profile of various miRNAs [15]. Another study combines LNA probes with enzyme-labeled fluorescence to detect average miRNA copy number per cell [16]. LNA probe with fluorescence in situ hybridization has been also used to detect miRNAs expression in frozen tissue sections [17]. In the present study, we demonstrated that MISH could be applied to evaluate miRNA expression level in paraffin-embedded archival tissue specimens, which makes large-scale study possible using TMA.

To demonstrate MISH can be explored to investigate miRNA expression in a high-throughput way, expression of miR-375 was detected in ESCC using a TMA containing 300 primary ESCC cases. The result indicates that MISH is a reliable approach to study miRNA expression in paraffin-embedded sections. Although MISH cannot quantify miRNA expression in individual cells, it is able to distinguish miRNA expression between tumor and surrounding non-tumor cells. In this study, informative miR-375 expression was detected in 249/300 (83%) of ESCC cases. Non-informative specimens were mainly caused by the samples loss. MISH result found that miR-375 was downregualted in 54.2% of ESCC tumor tissues compared with their paired non-tumor tissues. Clinical correlation study indicated that downregulation of miR-375 was significantly associated with lymph node metastasis (p = 0.04), advanced clinical stage (p = 0.003) and poor outcome (p = 0.04) in patients with ESCC. Multivariate COX analyses showed that miR-375 was not an independent risk factor for overall patient survival. These data demonstrate that MISH is a useful tool to evaluate miRNA status in a large-scale of samples by testing miRNA expression in paraffin sections. In addition, this approach may be also used to detect miRNA level in a single cell in blood and other body fluids. Further studies will be needed to develop this potential application of MISH assay.
